# Dr. Yeats's *Account of Ann Fooks's Case*

**Published:** 1814-01

**Authors:** G. D. Yeats

**Affiliations:** Bedford


*To the Editor of the Medical and Physical Journal.*

SIR,

I SHALL be obliged to you to insert in your next Number the subjoined case of Ischuria and Vomiting of Urine.

I am, Sir,

Your very obedient Servant,

*Bedford, Dec.* 10, 1813. G. D. YEATS, M.D.

*Bedford, Dec.* 6, 1811.—Ann Fooks, æt. 25, unmarried, about ten years ago, in the autumn of 1801, in jumping off a large log of wood, fell, and hit her stomach violently against a large stone, which did not give immediate pain, but caused a sensation of numbness from the back bone through the stomach. A degree of faintness supervened, which prevented her from rising immediately. This accident happened about two o'clock, one hour after having dined, solely on baked pudding, without having drank any thing. About two hours after the accident, during which she felt faint and languid, she became sick with pain at her stomach, and vomited up the whole of her dinner. The pain of her stomach, accompanied by great soreness on the slightest pressure, increased during that evening; and about ten at night she was again seized with vomiting, and brought up two tea-cupsful of blood, partly clotted, and of a dark color. She continued the whole of the night in great pain at the stomach, with repeated nausea, but without bringing up any thing. She took some gruel during the night, but it was brought up again in about an hour. On the following morning she again vomited a small tea-cupful of blood, more fluid, and not so dark-colored. She did not take any thing during the day from constant vomiting, attended occasionally with blood. This state of vomiting continued for five days, during which time she only took a little gruel as often as she could, for it very seldom remained on her stomach. At this period she sent for the medical attendant on the parish, who gave her some powders of a sour taste, which made her stomach smart, and they were thrown up. She still continued to be much troubled with the vomiting, bringing up blood more or less at different times. She was then ordered some purging salts, which remained on her stomach, and produced stools. The vomiting, however, still continued.

During the whole of this winter (1801-2) she took various medicines without any relief, the vomiting very frequently, almost daily, taking place, but only at times with blood. A troublesome cough also came on, which pained her stomach very much, attended with an expectoration of yellow phlegm, mixed with blood, and with occasional shiverings in the evening, succeeded by great heat at night, and a cold sweat towards morning. This cough and hectic state continued during the whole of the winter and spring, was relieved in the summer, and returned the following winter. She was so weak at this time as not to be able to go abroad, and was frequently confined to her bed. Towards the spring of 1802 the vomiting ceased for four or five weeks. The stomach, however, still continued very sore, and began to swell. After the five weeks had elapsed, the sickness and vomiting of blood returned, on which the swelling of the stomach subsided.

This state of occasional vomiting, with swelling and soreness of the stomach, continued for two years, viz. to 1804. At the end of this period (during which she never went farther from home than to take a short turn in the garden) she felt much better, so as to be able to walk some distance from home; but on her return the same day, which occupied three hours, from her being greatly fatigued, she felt great pain and soreness at the stomach, and on the following day the vomiting of blood returned, and continued for several days.* About two months after this, in the 15th year of her age, the menses made their appearance, although she was at the time very much troubled with the vomiting of blood, which continued two days after this flow of the menses ceased.†

For six years after this period, she continued in a varied state of vomiting, with soreness of the stomach, and great heaviness after taking any kind of food, as if a weight pressed upon the stomach. If the vomiting ceased for a time, the stomach swelled, and became hard. Four years after the

* The excitement of the system by the exertion of walking reproduced the vomiting of blood.

† I recollect the cases of two patients in the Infirmary who had vomiting of blood from mechanical injury. The vomiting of blood flowed in considerable quantity during the menstrual discharge. One of these patients had a suppression of urine for three days. In this state she walked to my house from home, a distance of three miles. She was sent to the Infirmary, where above two quarts of water were drawn off by the catheter. A third patient with suppression of urine, had also vomiting of blood. The coincidence of these cases with that of Fooks in the connection between suppression of urine and vomiting of blood, is curious. In Dr. Senter's case there was also vomiting of blood.

accident accident described in the beginning of the case, she fell down stairs, and received a violent blow on the breast, which deprived her of her senses for several hours; and on the following day she vomited blood as usual. For two days previous to this fall the vomiting had remitted, and the stomach was swelled, sore, and hard. From this accident the left breast became discoloured, and was swelled and painful, and continued more or less so for two years. During the whole of this time, that is up to 1809, from their commencement, the menses made their appearance, but not at exactly regular periods, and at all times when they flowed the vomiting of blood was considerable. Sometimes they would return in nine days. In September of this year was the last time they occurred.

In February, 1807, she was received into the Bedford Infirmary, under the care of one of the surgeons, the physician being absent in Ireland. At this time the vomiting was troublesome, occasionally with blood. The hectic state already described was also very distressing. She remained in the Infirmary about three months, during which a sore broke upon the breast and was healed, when she was dismissed with nearly the same symptoms as those with which she was received, save the curing of the breast.*

About a year after she came out of the Infirmary, I was desired to visit her. Her condition then was truly deplorable. I found her with a stomach much swelled and painful, and with almost constant vomiting of blood, sometimes dark, and sometimes of a lighter color. The tongue and internal parts of the lips and cheeks were dry, and of a black color; and there was an eruption in blotches of a similar kind, but not so black, spreading to the nose. She had frequent flushes of the face with the same hectic state already described. She was in every way a miserable object tormented with pain. For a few days I directed for her several articles of the materia medica without the smallest advantage. The potash in a state of effervescence, with the citric acid, caused an intolerable burning pain in the stomach, as if applied to an excoriated part. There is no doubt the repeated exertions of vomiting must have denuded the stomach of a considerable portion of the mucus which lines it, and is its natural protection against irritating substances. A great number of the internal vessels of the stomach were in an inflammatory state, and many of these vessels were ruptured, and were pouring out blood. These circum-

* A scar is visible on the breast.

E 2 stances stances very satisfactorily account for this extreme sensibility of the stomach. Indeed the appearance of what she vomited up, being of that dirty coffee color, similar to what is observed in the last stage of scirrhous stomach, impressed me strongly with the belief that there was an ulcerated surface within that viscus. In this miserable state, daily wasting from the exhausting influence of the discharge, and a preying hectic, it appeared probable that the powers of life could not long continue. I therefore discontinued all medicine, and desired her to live, as well as she was able to take it, on mucilaginous food. I occasionally called for about a month with the hope of finding an opportunity of rendering assistance, but none presented itself. I then ceased to visit her: it was indeed extremely painful to see her without having the power to afford relief to her complicated sufferings.

About Michaelmas, 1808, the previous summer having been passed in the state of vomiting already described, she began to complain of violent darting pains in the head, with indistinct and double vision; and the light was so painful to her eyes that it became necessary to hang a cloth before the window. This state lasted about six days, when she became insensible, and remained so for about ten days, sometimes dozing with her eyes half closed, moaning; and at times talking deliriously, and rolling her head about the pillow. During this period of delirious insensibility, a discharge of matter and blood, very offensive, took place one night from her ears and nose; and continued to discharge from her right ear for the space of six weeks. It was so considerable that it was necessary to renew the napkins which were placed under her head at first twice in twenty-four hours, although they were folded various ways at different times, in order to apply a clean surface to the head. Immediately after this discharge took place, the head became considerably relieved, and her senses began gradually to return. About a week after this discharge from the ear ceased, both arms, from the fingers to the shoulders, and both the lower extremities, from the feet to the hips, were attacked with considerable swelling of the œdematous kind, as they received the impression of the hands of any person who grasped her arms and legs. Her own hands were so enlarged and stiff with the swelling, that she could not close her fingers; and she was in a good deal of pain in both the upper and lower extremities. These swellings continued about three months, when they gradually subsided in the arms first. At Christmas, for two days and two nights, during this state, she passed not a drop of water, but there was a frequent irritation tation to make it.* A druggist in this place came to see her, and sent her a draught of the diuretic kind; and during the night she discharged her urine. During the time that the swellings prevailed the most, the greatest quantity of water she passed in twenty-four hours was about a pint, but generally less; and soon after the swellings began to subside, she states her urine to have been considerably increased. The swellings of the legs did not entirely subside till May. She does not recollect that during this period there was any thing remarkable in her stools, except that they were slimy; and she was very costive.

In September of this year, 1809, the vomiting ceased. Three months after this she was troubled with great hoarseness, which has since occasionally returned. About this period she felt a great deal better, the stomach gradually becoming easier, but never feeling well, and no swelling coming on.

About the beginning of the following June, 1810, while sitting in her chair, she was seized with giddiness and dimness of sight, followed by a paralytic affection of her left side, which lasted between three and four months, and then went off. The stomach was not swelled at the time, neither was there any vomiting, but some nausea. In July she was attacked with a strong shivering, succeeded by a cold sweat all over the body, and accompanied by a violent tetanic affection of the upper extremities, which were forcibly drawn upwards and towards the chest, attended with very great pain, and clenching of the hands. This painful state of spasmodic contraction continued four or five days. The cold sweat lasted several hours. During these four days she recollects nothing else in particular, except that she was extremely thirsty. She was quite sensible.

The food at this time kept down; and the following Michaelmas, 1810, she was removed down stairs from the bed-room, but sickness soon came on, and she was again carried up, when she vomited a considerable quantity of blood, which continued returning for a fortnight. This reduced her very much, and the vomiting then entirely ceased without any swelling coming on. The soreness of the stomach, however, continued No vomiting or swelling of the stomach again took place till about five weeks ago from this time (December 6, 1811), when the vomiting returned, but without blood.

* "In pain for want of making water, and all the time felt an inclination to make it, but could not," are her own words. A similar occurrence took place in Dr. Senter's case, some time before that suppression which immediately preceded the vomiting of urine.

For

For a week previous to this return of vomiting, she had past no urine.* For ten days after this suppression of urine, she had no stool naturally, although she took different kinds of opening medicine in the form of pills and mixture, and also some castor oil: the latter was rejected, but the pills remained. Twice during this period she vomited up fæculent matter. About two days after the second fæculent vomiting, she had a stool naturally, but extremely costive, hard, and lumpy, unmixed with any thing. With the effort of passing this stool, she had a violent pain just above the navel. After this she had no more fæculent vomiting, and had stools naturally, not, however, without the aid of medicines; and for several times they were costive, but afterwards they became more liquid. Soon after she had ceased to pass urine, the stomach again began to swell. It is now six weeks since she past any urine naturally; but it has frequently been brought up by vomiting, at the interval of one, or two, or three days, often clear, and unmixed with any thing. The last time she passed urine naturally was on the 21st of October, 1811. It was clear when first made, but became thick on standing. To the best of her recollection, the first time she vomited a fluid of a urinous taste was a day or two after she had the first costive stool,† and it was mixed with phlegmy slimy stuff; and from this period she has continued to vomit a nauseous fluid, sometimes clear like urine, and sometimes mixed with various matters, and says that her private parts and passages are hot, dry, and sore.‡ Since the return of the swelling of the stomach last mentioned, it has remained much swelled, painful, and hard; and for the last two days the vomiting is of the color of cho-

* At this suppression she described to me no symptom of irritation to pass her urine; and once or twice on visiting her, I discovered no protrusion or fullness such as would arise from a distended bladder, on examining the lower part of the belly, but pain was felt on pressure, and in the back it was a recurring symptom. It appeared to me to be that species of suppression which arises from an impeded flow of urine into the bladder from the ureters. "There is no tumor in the lower belly, nor desire to make water," says Cullen on this species. Nosol. p. 352. Some physiologists, however, are of opinion that other glands than the kidneys may secrete urine. Paper on Ischuria, p. 335.

† I think it very likely, that, with the fæculent vomiting, some urine was thrown up, not perceptible to the taste from the nauseousness of the former.

‡ Sometimes she has vomited urine twice in the same day, but generally at the intervals mentioned; but all the matters vomited at any time had a urinous taste.

colate colate grounds, of a very nauseous taste. Complains of much thirst; no appetite; the bowels are at present regular; is troubled with much shivering, and considerable fever and flushes of the face in the evening, and cold sweats towards morning; pulse 80, small, and weak; tongue clean, but redder than natural; says her mouth feels clammy; her tongue is sore, it is a little chopped in the middle; eyes light grey; hair dark brown; teeth very good, and perfectly white; skin fair, with a florid complexion before her illness, but now she is very pale; respiration quick and short, owing to full inspiration causing pain at the stomach; is also troubled with a cough; has very little rest; complains of a smarting, and darting pains in the stomach, which is swelled and prominent from the spleen to the liver, and to the umbilicus, protruding in an elevated state from those parts, and it is extremely tender to the slightest touch. The stomach does not wholly subside during the act of vomiting, but becomes hollow at the time at the upper part.* Such is the situation in which I saw this girl on the 6th of December, 1811.

*May* 8, 1812.—Since last report, she continued to vomit a urinous fluid until Sunday, April 12, at the interval generally of one or two days; and says, that the longer she continued without vomiting, the more nauseous and high-colored was the liquid vomited; for sometimes she would be three days without vomiting. On Monday, April 6, previous to the Sunday of passing her urine naturally, she took an emetic, which brought up by measurement nearly two quarts of a slimy liquid of a most nauseous urinous taste and smell. This emetic was taken about five P.M. This afforded her great relief from the harassing nausea and load at her stomach;

* This partial contraction of the stomach is a curious fact, to which, on another subject, Mr. Home, of the Hunterian school, alludes, in some experiments which he made. "During the investigation of the functions of the stomach, it was found, that while digestion is going on, there is a separation between the cardiac and pyloric portions, either by means of a permanent or muscular contraction. This fact placed the process of digestion in a new light, and led me to consider in what way the quantities of different liquors so often taken into the stomach can be prevented from being mixed with the half-digested food. Pursuing this inquiry, I found that the fluids are principally contained in the cardiac portion, and the food that has reached the pyloric portion is usually of one uniform consistence, so that the fluids beyond what are necessary for digestion would appear to be carried out of the stomach without ever reaching the pylorus." Phil. Trans. 1808.

bu but in the night, about twelve o'clock, she was seized with a coldness, and a great general shivering. This continued more or less the greatest part of the night. Upon the accession of this rigor, a very acute pain of the bowels, below the navel, came on, and was shortly succeeded by five or six evacuations per anum, of slimy clotted blood with tenesmus, unaccompanied by fæces.* "On the following morning (Tuesday) she became insensible, with muttering delirium, and she frequently screamed out from pain. She seemed stupid, and only appeared awake by her talking. Her eyes were in a great measure closed, except she was teased to be spoken to. When roused from this stupor, she would talk wildly, at times complaining of her back. This state lasted till the Sunday following, in the afternoon of which day she became more restless and uneasy, complaining of great pain in the lower belly, and about the loins. Her mother imagining from these circumstances that she wanted an evacuation, took her out of bed for the purposes of nature, when she past about a pint of dark-colored urine, of a strong smell, with great pain. After being put into bed again, her senses began gradually to return, and all delirious insensibility disappeared on the next morning (Monday), but she was confused at times during the night."† She now makes water naturally, but always with pain, and so little at a time, that she is three hours in making about a pint, the pan being under her all that time. It is now half past twelve meridie, and at the moment I am taking from herself the notes of her case, the pan is withdrawn, and she has past about a pint of a dark-colored fluid, of a very strong urinous smell, evidently mixed with blood. The general constitutional symp-

* Urine may have past off with the fæces at this time. The stomach never entirely subsided during the time of the suppression. It formed a reservoir for the reception of morbid secretions of its own disease, as well as of the urine. I may here mention, that in the Phil. Trans. for 1808 and 1811, some curious experiments are published by Mr. Home, which show a more direct communication between the stomach and urinary bladder, than through the medium of the general circulation. These experiments were made on dogs; in some the thoracic duct, in others the lower orifice of the stomach, was so well secured by ligature, that after the death of the animal it was clearly seen that nothing could pass that way; yet the urine was found highly tinged with rhubarb, an infusion of which had been injected into the stomach of the living animals after the ligatures were made,

† The account within the inverted commas was received from the mother and an attendant who were with her, as the girl herself does not know what happened to her during this state of insensibility.

 toms toms and local ones of the stomach continue as already described. She always feels pain and a burning at the stomach after any kind of food, which consists of sago, arrow-root, &c. These are generally taken with sugar, and sometimes with two tea-spoonfuls of cowslip wine. She has taken a pap-spoonful of port wine mixed with three parts of a cup of water; even thus diluted, it feels hot to the stomach. She cannot touch any thing of the kind except largely diluted, as it causes excruciating pain in the stomach. She takes her mucilaginous food in the quantity of a gill six times in twenty-four hours.

July 3, 1812.—She has continued to pass her urine naturally, which at first was mixed with blood; but within the last fortnight her urine has become clearer. She says she is quite certain the bloody urinous fluid comes by the natural passage entirely, as she has frequently past it without stool, and she has no discharge of any kind from the uterus. She has generally a stool daily, sometimes only once in two days; and her stools are invariably mixed with a bloody frothy matter, and sometimes the discharge is without fæces. The swelling of the stomach has subsided gradually after the bloody stools commenced, but the region of that viscus continues very sore to the touch, and gives great pain on pressure. She is unable to take any solid animal food without great distress: she swallowed a few days ago a small piece of roasted lamb, but she felt great weight and violent pain at her stomach for some hours, with nausea, when she was relieved by a stool similar to those already described. The only food she is able to bear is light soft pudding, arrow root, and things of that sort. Her appetite is very deficient; thirst considerable.

July 6.—Her nights are generally sleepless, on account of an aching uneasy feel at her stomach. Pulse about 86. I have this day minutely examined the whole of the abdomen. A tenderness is complained of above the pubes, extending a little way right and left. A tenderness is also felt over the liver and spleen; and upon pressure being made between these organs, exquisite soreness is complained of, and if the pressure be made with firmness, it causes agony with nausea. She is very pale and thin. Her breasts have not suffered the same degree of emaciation with the rest of her body; they are, however, flaccid. With a view of endeavoring to render her service, I directed a decoction of bark in almond milk, that the bark alone might not irritate the stomach, so highly sensible from disease. After being used for a while, it was given up, as it produced no great good, although she thought it improved her appetite. Opiate pills were directed at the same time to allay pain. She remained comparatively at ease from the use of the pills till the evening of July 22d, when she was seized with a cold shivering and pain in her limbs, succeeded by a burning heat all over her, accompanied with vertigo and dimness of sight. She has been quiet as to nausea and vomiting, till the accession of the cold fit, when she was very sick, and has since continued more or less so. The bowels are torpid, and she has recourse to an opening mixture. Her stools are of the same kind as those already described: The urine is of a natural appearance: she has now no pain in making it. The pulse is quick; much thirst; no disposition for food; stomach very tender to the touch, and not swelled.

Wednesday, August 26.—The stomach was a little swelled, and, as usual, sore to the touch on Sunday morning last. I saw her in this state. During Sunday night very great darting pains were felt in the stomach; sickness, but no vomiting, was troublesome; and Monday morning the swelling began to subside, followed by a considerable quantity of blood by stool; and the swelling is now quite gone down. Nausea continues at times.

Friday, August 28, twelve meridie.—I am now witnessing her vomiting blood: it is rather florid, without clots, and unmixed with any thing except phlegm. She has been sick the whole night; takes cold water with relief.

29th.—The vomiting of blood ceased yesterday afternoon, but returned this morning with much violence. It is now eight P. M. I have seen her four times to-day, during which she has been nearly in a constant state of vomiting, and has brought up by measurement, with what she vomited yesterday, nearly a quart of blood, to all appearance pure. Her pulse is exceedingly quick and feeble, and at one time to-day thready. She has the appearance of a dying person. At one of my visits to-day were present Messrs. Short, Gibbon, and Leach, surgeons.* At my visit at noon she was directed to take some Tinct. Opii in Lac. Amygdal; it had no effect in checking the vomiting. Her constant call is for cold water, from which she finds the most relief: one grain of opium to be taken to-night.

Sunday, August 30.—She took the opiate pill last night about nine o'clock. From that time till four o'clock this

* At this meeting she was considered in a very alarming state, and too hazardous to remove her to the Infirmary at the time, as I had intended. I abandoned this intention from the alarming state in which she continued, showing, beyond the possibility of a doubt, a highly morbid condition.

morning morning there were intervals of rest. At four the vomiting returned; eleven A. M. sickness and retching still continue. She has brought up nearly half a pint more of blood since I saw her last night about eight. Complains of being very faint; constantly calls for cold water; has taken since the day before yesterday nothing more, the cold water excepted, than half a tea-cupful of sago. Pulse 118, feeble. About one hour ago she past, without pain, about half a pint of urine, pale, like what hysteric people pass. Repeat the opiate at six o'clock P. M. Since my visit at eleven to-day, she has vomited nearly half a pint more of blood. The vomiting has ceased since five, but there is a gulping or retching without bringing up any thing. Complains of dimness of sight, and much pain in the lower limbs. On examination, the right leg is drawn up in a contracted state: it requires some force in attempting to straighten it; the tendons are rigid. While sitting here she has past a gill and a half of urine, limpid, but of a urinous smell. Repeat the pill.

August 31.—Past a restless night from sickness, but the hæmatemesis has much diminished; blood, however, is occasionally brought up. Last evening the left arm was convulsed for a time similar to the leg. The body being costive, an enema of oil, sugar, and gruel was directed last night, to be given this morning if no stool was past. One came away in the night very watery, and so quick that there was no time to give the pan. At four and five this evening she past two stools, fœtid, loose, and without blood. Nausea is still troublesome, but she took a tea-cupful of mutton broth, which remained. Has just made water: the pan was introduced in my presence, and withdrawn, containing somewhat more than a gill of limpid urine.

Nov. 8.—Since the report taken on the 31st of August, her symptoms have been nearly the same as those already described. She has at times vomited much blood; and instead of the opium alone, she was directed to take every six hours pills containing one grain of opium with five of gum kino. These had no effect in permanently restraining the hæmorrhage. To allay the heat of the stomach attending this symptom, she has constant recourse to cold water. She complains to-day of considerable pain over the region of both kidnies, which are tender on pressure. Pulse very quick, small, and feeble. Complains of being faint; and she has just broke out into a sweat. She has fainted while I am writing. Pulse at the wrist obliterated; and she is motionless, with the interruption of an occasional sob and deep sighing. This faintness lasted about three minutes, when it

 went went off, preceded by yawning, and the gradual return of the pulse at the wrist. Has suffered several such fainting fits since the return of the vomiting of blood, which was witnessed by Mr. Short, Mr. Gibbon, Mr. Leach, and myself, at that time. The pills do not give the ease they first did. She was ordered a phial of laudanum, to be had recourse to in the quantity that circumstances might require.

January 11, 1813.—Since the last report in November, the symptoms of vomiting, soreness of stomach, &c. have continued in varied degrees. During this interval, I have visited her many times, and have seen her vomit much blood. Her urine has been past naturally, and almost always pale. On visiting her to-day I find her with a voice almost inaudible from great hoarseness. The whole of the left side is spasmodically affected. The fore-arm is so strongly contracted that with much force I could not straighten it. The left leg is drawn to an acute angle with the thigh, by the spasmodic state of the muscles. Her pulse is exceedingly quick; the tongue drier than usual; the countenance extremely pale; says her stomach feels less sore. She has vomited a dark-colored fluid with a tenacious thick sediment, exactly of the color of chocolate.

Sunday, June 20, 1813.—I have very frequently visited this girl since I last took an account. I have uniformly found her in the same state of painful disease and misery. The pulse is on this day 120, and feeble; countenance very pale; tongue very dry. The quantity of food taken lately is very small, chiefly tea; but it is frequently thrown up again, and sometimes with blood. The ankle and instep have swelled lately, and are so at present, indenting on pressure.

Nov. 19, 1813.—Such is the history of the case, copied from my notes, which were taken entirely from the girl herself, except those parts of which, from insensibility, she could not give an account. It was read over to her this evening in the presence of a clergyman, as being the statement I had taken from herself. It was not published before at the call that was made, for one reason, which was given to Mr. Pulley in conversation before that call appeared in print, viz. that I waited to see whether death would take place, that I might add the dissection (which I was promised) to the history of the symptoms. The girl is now as she has been at different periods of her illness, in a comparatively meliorated state. She is still confined to bed; is very pale and thin. The vomiting of blood is much mitigated, though she brings up a small quantity occasionally. The bowels are torpid; urine very pale; no menstruation; it has ceased for a considerable time, time. She is not able to eat meat in a solid form.* She occasionally takes some broth,—that but seldom; lives chiefly on farinaceous food, and puddings made of apple; is very thirsty. The region of the stomach is more prominent than natural, and it is sore to the touch. For several weeks during the last three months she sweated so profusely that it was often necessary to have her linen changed every hour.

[This valuable paper will be concluded in our next Number.]

* She has not ventured upon any since the great suffering she experienced from the small piece she took as reported on the 3d of July. Dr. Girdlestone's patient was also unable to take meat.

